# The Product of Matrix Metalloproteinase Cleavage of Doxorubicin Conjugate for Anticancer Drug Delivery: Calorimetric, Spectroscopic, and Molecular Dynamics Studies on Peptide–Doxorubicin Binding to DNA

**DOI:** 10.3390/ijms21186923

**Published:** 2020-09-21

**Authors:** Kamila Butowska, Krzysztof Żamojć, Mateusz Kogut, Witold Kozak, Dariusz Wyrzykowski, Wiesław Wiczk, Jacek Czub, Jacek Piosik, Janusz Rak

**Affiliations:** 1 Laboratory of Biophysics, Intercollegiate Faculty of Biotechnology University of Gdańsk and Medical University of Gdańsk, Abrahama 58, 80-307 Gdańsk, Poland; jacek.piosik@biotech.ug.edu.pl; 2 Department of Physical Chemistry, Faculty of Chemistry, University of Gdańsk, Wita Stwosza 63, 80-308 Gdańsk, Poland; davelombardo@wp.pl (W.K.); janusz.rak@ug.edu.pl (J.R.); 3 Department of General and Inorganic Chemistry, Faculty of Chemistry, University of Gdańsk, Wita Stwosza 63, 80-308 Gdańsk, Poland; krzysztof.zamojc@ug.edu.pl (K.Ż.); dariusz.wyrzykowski@ug.edu.pl (D.W.); 4 Department of Physical Chemistry, Faculty of Chemistry, Gdańsk University of Technology, Narutowicza 11/12, 80-233 Gdańsk, Poland; giggsmk@op.pl (M.K.); jacek.czub@pg.edu.pl (J.C.); 5 Department of Biomedical Chemistry, Faculty of Chemistry, University of Gdańsk, Wita Stwosza 63, 80-308 Gdańsk, Poland; wieslaw.wiczk@ug.edu.pl

**Keywords:** doxorubicin, matrix metalloproteinases, intercalation, DNA, cleavable peptide, drug delivery

## Abstract

Matrix metalloproteinases (MMPs) are extracellular matrix degradation factors, promoting cancer progression. Hence, they could provide an enzyme-assisted delivery of doxorubicin (DOX) in cancer treatment. In the current study, the intercalation process of DOX and tetrapeptide–DOX, the product of the MMPs’ cleavage of carrier-linked DOX, into dsDNA was investigated using stationary and time-resolved fluorescence spectroscopy, UV-Vis spectrophotometry and isothermal titration calorimetry (ITC). The molecular dynamics (MD) simulations on the same tetrapeptide–DOX^…^DNA and DOX^…^DNA systems were also performed. The undertaken studies indicate that DOX and tetrapeptide–DOX can effectively bond with dsDNA through the intercalation mode; however, tetrapeptide–DOX forms less stable complexes than free DOX. Moreover, the obtained results demonstrate that the differences in DNA affinity of both forms of DOX can be attributed to different intercalation modes. Tetrapeptide–DOX shows a preference to intercalate into DNA through the major groove, whereas DOX does it through the minor one. In summary, we can conclude that the tetrapeptide–DOX intercalation to DNA is significant and that even the lack of non-specific proteases releasing DOX from the tetrapeptide conjugate, the presence of which is suggested by the literature for the efficient release of DOX, should not prevent the cytostatic action of the anthracycline.

## 1. Introduction

Cancer—the second leading cause of death worldwide—can be treated by several modalities, of which chemotherapy represents 36% of the treatment options [1,2]. Anthracyclines are one of the most widely used chemotherapeutic drugs for the treatment of various types of cancer including leukemia, melanoma, Kaposi’s sarcoma, and solid tumors, e.g., breast or prostate [3,4]. Doxorubicin (DOX) is one of the well-known anthracycline antibiotics, which was isolated in the 1960s from a mutated variant of *Streptomyces peucetius* (var. *S. caesius*) [5]. It is an important chemotherapeutic agent with a wide spectrum of activity [3,6,7]. The mechanism of DOX action is related to its intercalation into cellular DNA leading to the inhibition of the synthesis of macromolecules, DNA cross-linking, and alkylation, as well as the generation of free radicals and the inhibition of topoisomerase II [8,9,10]. Despite its clinical efficacy, it has severe disadvantages, including cardiotoxicity and myelosuppression [11,12]. Moreover, DOX can lead to several side effects that mainly affect the brain, kidneys, and liver [13]. 

One of the strategies to increase therapeutic efficiency, with high binding specificity and reduced toxic effects of a drug, is to conjugate it with polymers, lipid nanoparticles, or antibody–drug conjugates [14,15,16]. Such an approach can lead to a system characterized by enhanced solubility, permeability, and retention in body plasma that triggers drug release in cancer cells [17]. A variety of polymers, including polymer–drug conjugates (PDCs), polymer–protein/peptide, polymer–DNA, and their hybrids, have been developed in the past decades as selective therapeutic agents. Several examples of PDCs have been observed to couple a hydrophobic drug with hydrophilic polymers such as poly(lactic-co-glycolic acid) (PLGA) and poly(ethylene glycol) (PEG) [18,19]. However, PDCs with increased stability, solubility, and lower toxicity would not be suitable for cancer therapy if they did not release an anticancer component at the target place. To overcome this problem, PDCs release a drug using a peptide fragment selectively cleaved by enzymes, or under specific conditions [20]. Matrix metalloproteinases (MMPs) are a group of over 20 proteolytic enzymes characterized by their ability to remodel and degrade the extracellular matrix [21]. MMPs are overexpressed in newly formed tumor tissues, thereby promoting angiogenesis, and are involved in cancer progression; MMP-2 and MMP-9, in particular, play a critical role in this process [22,23]. Therefore, MMPs could constitute a promising strategy for targeted therapeutic agent delivery via an enzyme-triggered release mechanism [24]. According to the literature, there are a series of substrate peptide sequences degradable in the presence of MMPs, e.g., Gly–Pro–Leu–Gly–Ile–Ala–Gly–Gln, Pro–Val–Gly–Leu–Ile–Gly, or Pro–Leu–Gly–Val–Arg, where cleavage occurs between leucine (Leu) and glycine (Gly) [25,26,27]. Using this approach, new polymer–drug conjugates with a selective peptide sequence between the carrier (polymer) and the cargo (drug) have been designed [28]. The drug release from enzymatically cleavable prodrugs occurs within a two-step mechanism: (1) a prodrug is cleaved by MMPs and is (2) further cleaved or hydrolyzed to the desired drug [29]. Hence, some reports suggest that additional proteases are important for liberating a free drug from intermediate cleavage products [30]. 

**Figure 1:**
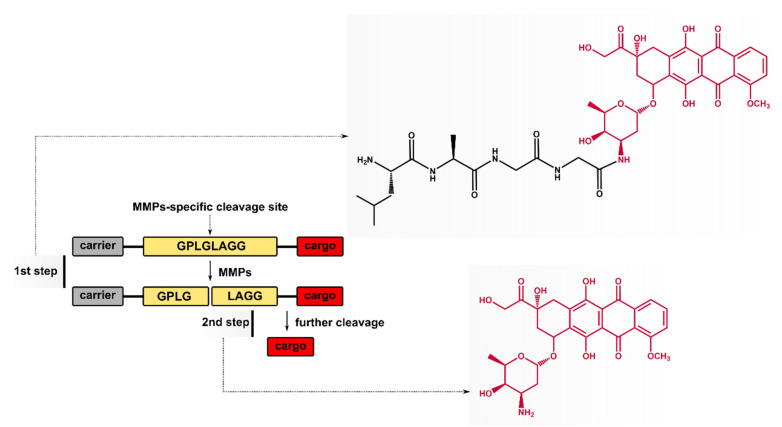
Doxorubicin (DOX) release strategies for matrix metalloproteinase (MMP)-cleavable prodrugs.

In this study, we present the synthesis of a tetrapeptide–DOX conjugate (Leu–Ala–Gly–Gly–DOX, i.e., 4-pep–DOX), as a potential result of MMPs’ action on the substrate peptide sequence (Figure 1). The selected MMP-2/-9-cleavable peptide, containing the Leu–Gly sequence, was expanded to Gly–Pro–Leu–Gly–Leu–Ala–Gly–Gly, which is cleavable by the chosen MMPs’ domain [31]. We demonstrated the impact of the presence of the tetrapeptide fragment in 4-pep–DOX on its intercalating properties to dsDNA, and compared the tetrapeptide–DOX^…^dsDNA complex with free doxorubicin, namely DOX^…^dsDNA. The characteristics of the dsDNA complex with 4-pep–DOX and DOX were studied by spectrophotometric titration, steady-state, and time-resolved fluorescence spectroscopy, as well as isothermal titration calorimetry (ITC). Furthermore, molecular dynamics (MD) simulations were carried out to determine the preferred binding sites to dsDNA. To the best of our knowledge, this is the first report where an MMP-cleavable product (Leu–Ala–Gly–Gly–DOX) has been characterized at the molecular level.

## 2. Results and Discussion

### 2.1. Cleavage of Peptide by MMPs

**Figure 2:**
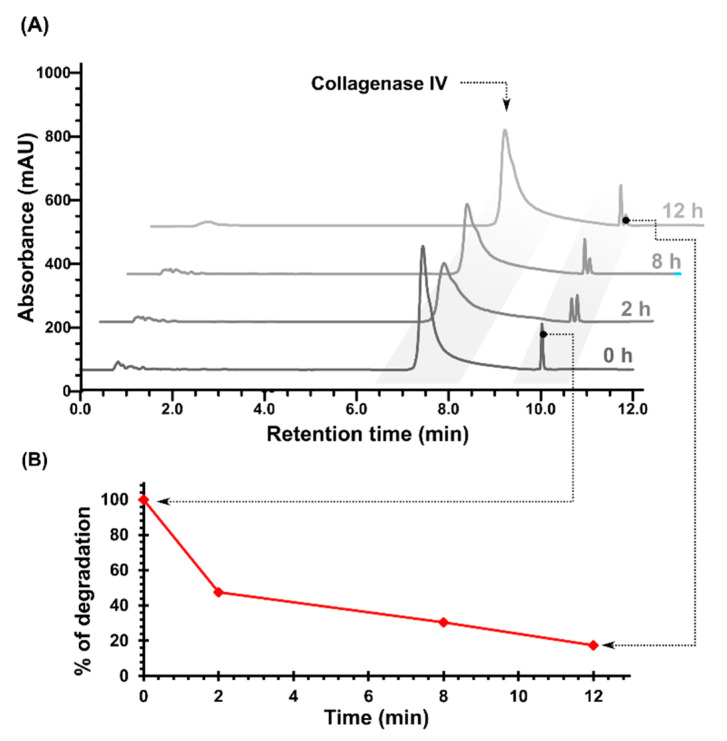
(**A**) HPLC chromatograms of the MMP-2/-9-cleavable peptide after 2, 8, and 12 h of incubation with collagenase IV. (**B**) The curves depicting the efficacy of enzymatic cleavage, triggered in a time-dependent manner.

The MMP-2/-9-cleavable peptide was degraded by collagenase IV (containing MMP-2/-9) in a time-dependent manner. The MMPs’ substrate specificity, revealed by the MS analysis, is consistent with that reported previously and indicates that the proposed octapeptide can be responsive to MMPs [32,33]. The MALDI TOF spectrum proved that the octapeptide was cleaved between leucine (Leu) and glycine (Gly), yielding two products, namely Fmoc–Gly–Pro–Leu–Gly and Leu–Ala–Gly–Gly (see Figure S1, Supplementary Materials). Consistently, the HPLC chromatogram (Figure 2A) shows two peaks from the enzymatic digestion—the substrate, Fmoc–Gly–Pro–Leu–Gly–Leu–Ala–Gly–Gly, and the product, Fmoc–Gly–Pro–Leu–Gly, at 10.30 and 10.04 min, respectively. During incubation, the Fmoc–Gly–Pro–Leu–Gly peak significantly increased, whereas the octapeptide peak disappeared. After 12 h of incubation, 17.43% of the substrate and 82.42% of the MMP-2/-9 cleaved product were observed. In order to investigate how fast the octapeptide cleaved, the aliquots were removed after 2, 8, and 12 h. After 2 h of incubation, 52.54% of Fmoc–Gly–Pro–Leu–Gly was detected and after 8 h it increased to 69.53% (Figure 2B). The efficiency of the enzymatic digestion reached 82.42% after 12 h of incubation. The results proved that the octapeptide can be cleaved by collagenase IV and that the amount of product formed increases with the incubation time.

### 2.2. Synthesis of Tetrapeptide–DOX

**Figure 3:**
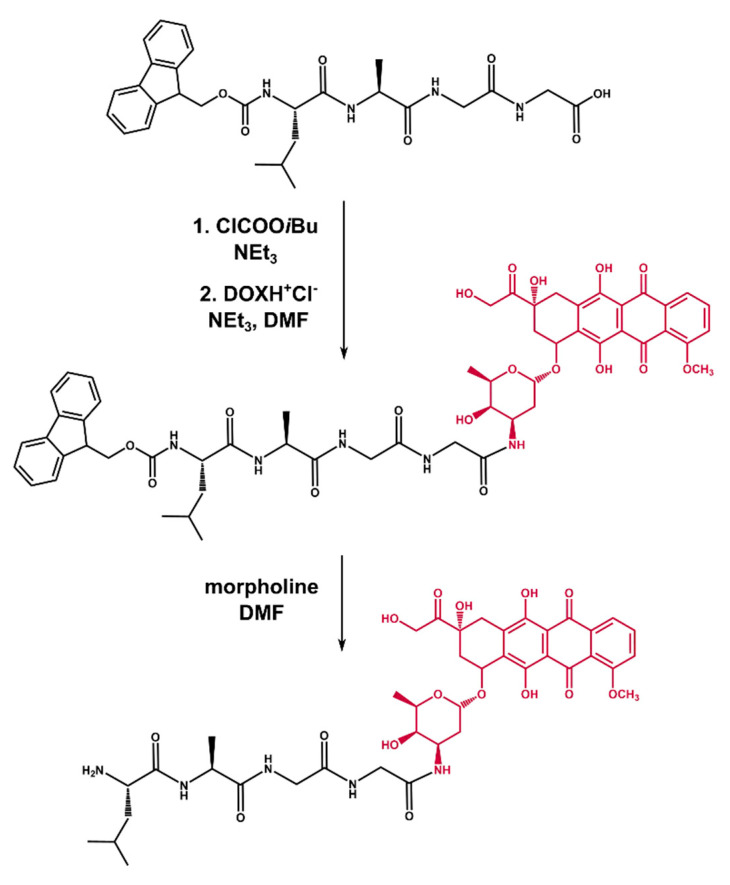
Synthesis scheme of the Leu–Ala–Gly–Gly–DOX conjugate.

In order to synthesize the desired conjugate, we performed a two-step procedure (Figure 3). First, Fmoc–Leu–Ala–Gly–Gly was coupled with DOX using the mixed anhydride method. For this purpose, Fmoc–Leu–Ala–Gly–Gly was treated with isobutyl chloroformate in the presence of triethylamine in DMF, followed by the addition of DOX (obtained independently by the reaction of DOX hydrochloride with TEA in DMF). The so-obtained raw Fmoc–Leu–Ala–Gly–Gly–DOX was purified by preparative column chromatography. In the second step, Fmoc–Leu–Ala–Gly–Gly–DOX was deprotected with a 50% morpholine solution in DMF, furnishing raw Leu–Ala–Gly–Gly–DOX, which was finally isolated with the HPLC system and proved by MALDI TOF/TOF (see Figure S2, Supplementary Materials). It is worth mentioning that we had tested two different deprotection methods with 1,8-diazabicyclo[5.4.0]undec-7-ene (DBU) and piperidine, respectively. In both cases, we struggled through some difficulties. As far as DBU is concerned, dibenzofulvene is released during the reaction, which readily reacts, as an electrophile, with N-terminal amino acid residue leading to undesired by-products [34]. When it came to piperidine, we obtained the desired product in a very poor yield, which could be due to the presumed dealkylation properties of piperidine (DOX possesses a methoxyl function in its constitution) [35].

### 2.3. Spectrophotometric Titrations

**Figure 4:**
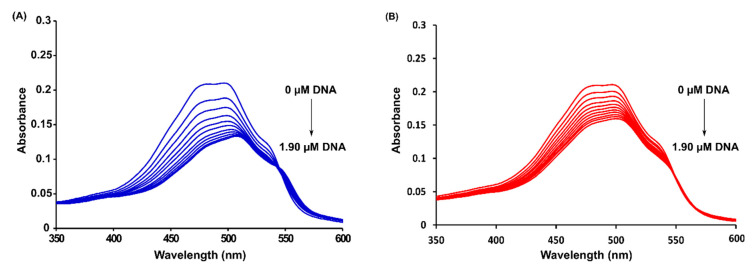
UV-Vis absorption spectra of DOX (**A**) and 4-pep–DOX (**B**) in the presence of dsDNA at various concentrations in 10 mM Tris-HCl buffer (pH 7.2) at 25 °C (the initial concentration of DOX and 4-pep–DOX = 18 µM).

In order to evaluate the binding strength of DOX and 4-pep–DOX to dsDNA, the binding/association constants (K_A_) of the resulting complexes were determined from the changes in absorbance of DOX and 4-pep–DOX bands with increasing amounts of dsDNA (Figure 4). The intrinsic association constants (K_A_) of the complexes were calculated according to the Benesi–Hildebrand equation:

(1) $$\frac{A_{0}}{A-A_{0}}=\frac{\varepsilon_{f}}{\varepsilon_{b}-\varepsilon_{f}}+\frac{\varepsilon_{f}}{\varepsilon_{b}-\varepsilon_{f}}\cdot \frac{1}{K_{A}\left[\mathrm{DNA}\right]},$$

where A_0_ and A are the absorbances of DOX/4-pep–DOX in the absence and presence of dsDNA in the solution, respectively, ε_f_ and ε_b_ correspond to the extinction coefficients of free and bound ligands, respectively, while [DNA] is the concentration of dsDNA. The ligand–dsDNA binding constants were calculated from the ratio of the intercept to the slope of the linear equation of the plot of $\frac{A_{0}}{A-A_{0}}$ (measured at 480 nm) to $\frac{1}{\left[\mathrm{DNA}\right]}$, and were determined to be K_A DOX_^…^_dsDNA_ = 1.10 × 10^6^ M^−1^ and K_A 4-pep–DOX_^…^_dsDNA_ = 0.54 × 10^6^ M^−1^. The free energies of the binding (ΔG) were calculated using the standard thermodynamic relationship, ΔG = −RTlnK_A_, and were determined to be ΔG_DOX_^…^_dsDNA_ = −8.23 kcal/mol and ΔG_4-pep–DOX_^…^_dsDNA_ = −7.81 kcal/mol.

### 2.4. Steady-State and Time-Resolved Spectroscopy

**Figure 5:**
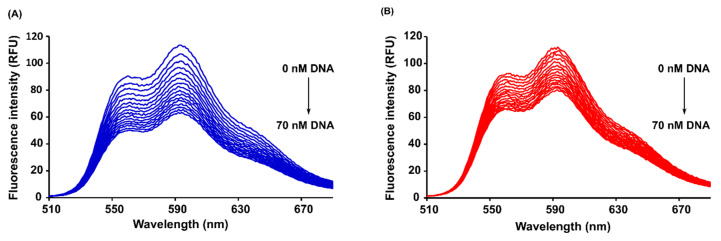
Fluorescence emission spectra of DOX (**A**) and 4-pep–DOX (**B**) in the presence of dsDNA at various concentrations in 10 mM Tris-HCl buffer (pH 7.2) at 25 °C (the initial concentration of DOX and 4-pep–DOX = 1.94 µM).

Figure 5 shows the fluorescence emission spectra of DOX and 4-pep–DOX recorded in the presence of various dsDNA concentrations, using drug excitation at 490 nm. It can be observed that the fluorescence intensity of both drugs decreases regularly with the increasing concentration of dsDNA, which may result from a variety of processes such as excited state reactions, ground-state complex formations, or collisional processes.

**Figure 6:**
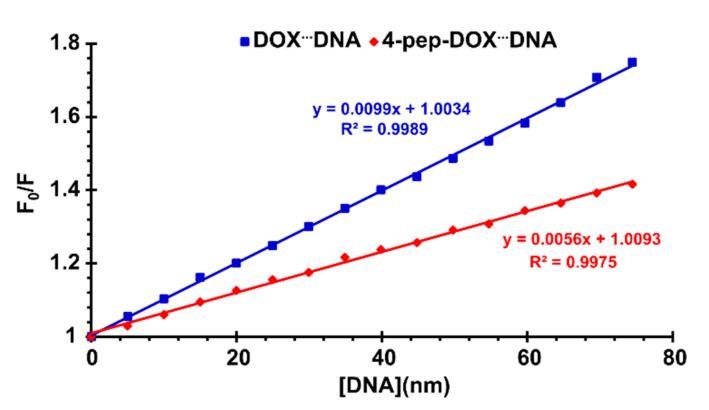
Stern–Volmer plots for the DOX^…^dsDNA and 4-pep–DOX^…^dsDNA systems in 10 mM Tris-HCl buffer (pH 7.2) at 25 °C.

In order to confirm the quenching mechanism, the fluorescence quenching was analyzed according to the Stern–Volmer equation:

(2) $$\frac{F_{0}}{F}=1+K_{SV}\left[Q\right]=1+k_{q}\tau_{0}\left[Q\right],$$

where *F*_0_ and *F* are the fluorescence intensities in the absence and presence of quencher, [*Q*] is the quencher concentration, *K_SV_* is the Stern–Volmer quenching constant, *k_q_* is the bimolecular quenching rate constant, while *τ*_0_ is the lifetime of the fluorophore in the absence of quencher [36]. The graphs of $\frac{F_{0}}{F}$ versus [*Q*] plotted according to the Stern–Volmer equation are shown in Figure 6. It can be observed that straight lines were obtained for both drug–DNA complexes, indicating that, in the investigated range of concentrations, the quenching is purely static or dynamic. Since the *τ*_0_ values for free DOX and 4-pep–DOX around neutral pH were determined to be 1.057 ns and 1.076 ns, respectively (see below), the values of the bimolecular quenching rate constants (*k_q_*) are 9.37 × 10^15^ M^−1^ s^−1^ for DOX^…^dsDNA and 5.20 × 10^15^ M^−1^ s^−1^ for 4-pep–DOX^…^dsDNA complexes. As these values are much larger than the maximum scatter collision quenching constant (2.0 × 10^10^ M^−1^ s^−1^) [37], this indicates that the fluorescence quenching effects of DOX and 4-pep–DOX by dsDNA are not initiated by dynamic collisions, but rather caused by the formation of ground-state complexes.

Given that the fluorescence quenching of both DOX and 4-pep–DOX by dsDNA is static, the dissociation constants (*K_D_*) of the newly formed complexes can be determined by the Lineweaver–Burk formula [38]:

(3) $$\frac{1}{F_{0}-F}=\frac{1}{F_{0}}+\frac{K_{D}}{F_{0}\left[0\right]}$$

**Figure 7:**
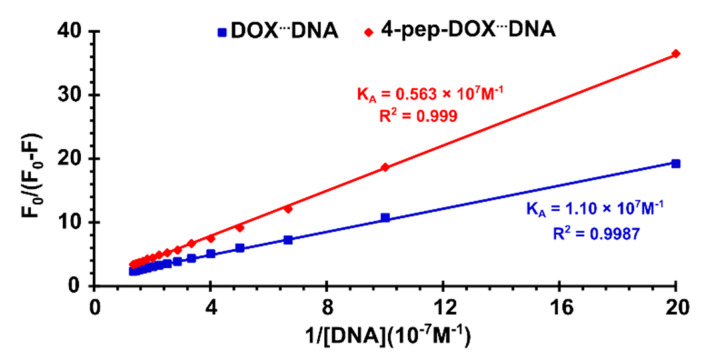
Lineweaver–Burk double-reciprocal curves of $\frac{F_{0}}{F_{0}-F}$ vs. $\frac{1}{\left[\mathrm{DNA}\right]}$ for the DOX^…^dsDNA and 4-pep–DOX^…^dsDNA systems in 10 mM Tris-HCl buffer (pH 7.2) at 25 °C.

The Lineweaver–Burk double-reciprocal plots were constructed based on the relationship of $\frac{F_{0}}{F_{0}-F}$ vs. $\frac{1}{\left[\mathrm{DNA}\right]}$ (Figure 7).

From the regression analysis, the association constants ($K_{A}=K_{D}^{-1}$) between dsDNA and both studied drugs were determined to be K_A DOX_^…^_dsDNA_ = 1.10 × 10^7^ M^−1^ and K_A 4-pep–DOX_^…^_dsDNA_ = 0.56 × 10^7^ M^−1^ (as the averages of two independent experiments), respectively. The observed discrepancies between the K_A_ values obtained from spectrophotometric and spectrofluorometric titrations may result from the specificity of each method, as well as from differences in the experimental conditions (i.e., concentrations). By comparing the measured association constants, it is clear that both DOX and 4-pep–DOX show a high affinity to dsDNA; however, the complexes formed by DOX are slightly more stable compared to those of 4-pep–DOX. When small molecules bind independently to a set of equivalent sites on a macromolecule, the number of binding sites (*n*) can be found from the following Scatchard equation [39]:

(4) $$\log\left[\frac{F_{0}-F}{F}\right]=\log K_{A}+n\log\left[Q\right],$$

**Figure 8:**
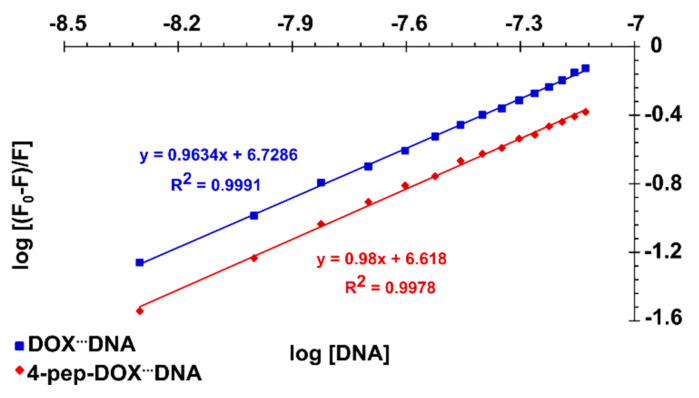
Plots of $\log\left[\frac{F_{0}-F}{F}\right]$ vs. log[DNA] for the DOX^…^dsDNA and 4-pep–DOX^…^dsDNA systems in 10 mM Tris-HCl buffer (pH 7.2) at 25 °C.

The plots of $\log\left[\frac{F_{0}-F}{F}\right]$ vs. log[*Q*] for both DOX and 4-pep–DOX are shown in Figure 8. It can be observed that the *n* values, determined as the slopes of the straight-line plots, were found to be approximately 1.0 for both ligands that are bound per dsDNA molecule. Furthermore, the association constants estimated by this method are basically in accordance with those obtained by the Lineweaver–Burk equation (K_A DOX_^…^_dsDNA_ > K_A 4-pep–DOX_^…^_dsDNA_).

The determination of fluorescence lifetimes was carried out to confirm that the static quenching is the sole reason for the observed fluorescence quenching. According to the performed time-resolved experiments, the fluorescence lifetimes of DOX and 4-pep–DOX were constant, regardless of the dsDNA concentration, and equal to 1.057 ± 0.022 ns for DOX (the value is in good agreement with literature data [40]) and 1.076 ± 0.006 ns for 4-pep–DOX. Therefore, *τ*_0_/*τ* = 1, which is characteristic for pure static quenching. This is in a good agreement with the results presented in Figure 4, since the formation of a complex is often reflected in a change of the absorption spectrum of the fluorophore.

### 2.5. Isothermal Titration Calorimetry

**Figure 9:**
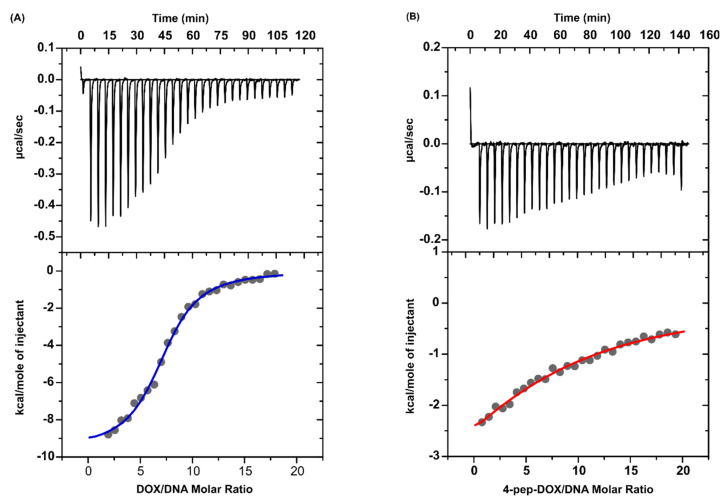
Calorimetric titration isotherms of the binding interaction between (**A**) DOX and dsDNA and between (**B**) 4-pep–DOX and dsDNA in 10 mM Tris-HCl buffer (pH 7.2, 25 °C).

The ITC method was applied to determine the binding constants (*K_ITC_*) and the thermodynamic parameters (Δ*G_ITC_*, Δ*H_ITC_*, Δ*S_ITC_*) for the interactions of DOX and 4-pep–DOX with dsDNA. Representative binding isotherms for the interactions under study are shown in Figure 9, and the thermodynamic parameters are summarized in Table 1. The molar ratios of the resulting complexes were estimated based on ITC data and they are 7.29 (±0.09) and 7.98 (±1.23) for DOX to dsDNA and 4-pep–DOX to dsDNA, respectively. The binding constants of the resulting complexes, as well as the binding enthalpies, were obtained directly from the calorimetric experiments by fitting binding isotherms (a nonlinear least-squares procedure) to a model that assumes a single set of identical binding sites.

The standard thermodynamic relationships, namely Δ*G_ITC_* = −RTln*K_ITC_* = Δ*H_ITC_* − TΔ*S_ITC_*, were used to calculate the free energy of binding (Δ*G_ITC_*) and the entropy change (TΔ*S_ITC_*). The formation of the investigated dsDNA complexes is an enthalpy-driven process (Table 1). The negative values of the binding enthalpy and small entropic contribution correspond to the intercalative nature of the tetracyclic core of the low-molecular ligands. The modification of the DOX structure with a Leu–Ala–Gly–Gly tetrapeptide results in a decreasing strength of interaction. This phenomenon is reflected in the values of the binding constant log*K_ITC_* (DOX–dsDNA) > log*K_ITC_* (4-pep–DOX–dsDNA) and remains in agreement with the results obtained from the spectroscopic measurements. In contrast to DOX, the presence of the hydrophobic peptide chain in the structure of 4-pep–DOX hinders the intercalating interactions of the modified ligand with dsDNA, and weakens its ability to form hydrogen-bonding and electrostatic interactions, which are typical for groove binders [41]. According to the general rule, the more new bonds are formed and the stronger they are, the more energy (heat) is released. Thus, the difference in the binding enthalpy results from the different mode of the DOX–dsDNA and 4-pep–DOX–dsDNA interactions. This phenomenon probably is the most important factor responsible for the release of a larger amount of energy in the case of DOX binding to dsDNA. 

### 2.6. Molecular Dynamics Simulations

**Figure 10:**
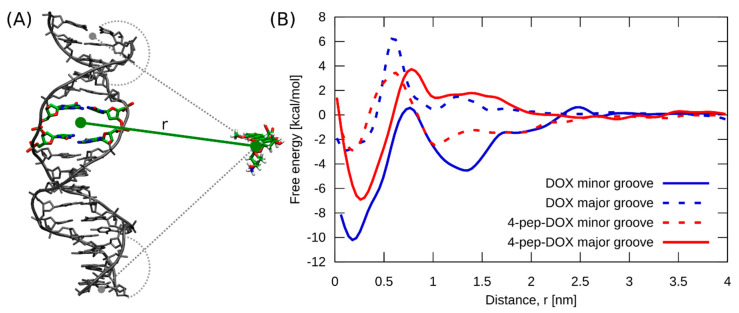
(**A**) Intercalation of DOX and 4-pep–DOX into dsDNA in the simulation was studied using the coordinate defined as the separation distance between the centers of mass of the two base pairs forming the intercalation site, and the planar chromophore of the ligand (green solid line). In addition, one-sided harmonic potentials were used to prevent association of the ligands at the dsDNA ends (see Methods section, grey dashed lines.) (**B**) Free energy profiles for the formation of the intercalation complexes of DOX and 4-pep–DOX with dsDNA as a function of the separation distance between them (r), in two intercalation modes (minor/major groove).

To provide a molecular-level understanding of the differences in the stability of the intercalation complexes formed by DOX and 4-pep–DOX with dsDNA, we computed the free energy profiles for the two different intercalation modes in which the amino sugar moiety is extended into either the minor or major groove. As a collective coordinate describing the intercalation process, we used the separation distance between the centers of mass of the two base pairs forming the intercalation site and the planar chromophore of DOX and 4-pep–DOX (see Figure 10A).

Comparison of the two free energy profiles obtained for the DOX^…^dsDNA system (blue lines in Figure 10B) strongly suggests that the highly stable DOX intercalation complex, observed in spectrophotometric titrations and ITC, corresponds to the minor-groove binding mode. Indeed, the intercalation of DOX from the major-groove side is 7.5 kcal/mol less favorable. This conclusion is further supported by a very good agreement between the depth of the main free energy minimum computed for DOX intercalating from the minor groove (−9.8 kcal/mol at ~0.2 nm) and the measured binding free energies. The free energy simulations further revealed that the intercalation of DOX is preceded by its relatively strong binding to the dsDNA surface (−4.5 kcal/mol) with a clear kinetic barrier to overcome. In contrast, 4-pep–DOX (red lines in Figure 10B) shows a 3.7 kcal/mol preference for intercalation from the major groove. The predicted absolute binding free energy in this case (−6.7 kcal/mol) is consistent with our ITC results and, to a lesser extent, the spectrophotometric values, supporting the conclusion that the stable intercalation complexes of 4-pep–DOX observed in our experiments are in fact characterized by the major-groove binding mode.

To structurally compare the four intercalation complexes considered in Figure 10B, we performed a cluster analysis of the MD-generated unbiased ensembles. This was done using the hybrid k-centers k-medoids clustering algorithm for all heavy atoms of DOX and 4-pep–DOX, with a root mean square deviation (RMSD) cut-off of 0.3 nm. Before clustering, the structures were superimposed by minimizing a heavy-atom RMSD for the two dsDNA base pairs forming the intercalation site [42].

**Figure 11:**
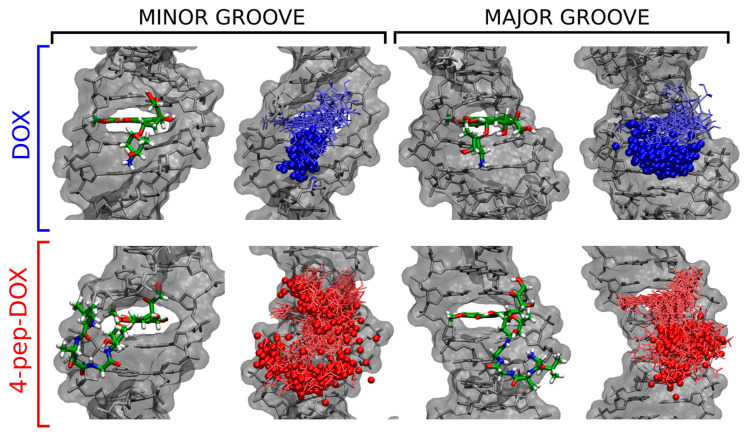
The most likely structures (**left**) and the range of ligand fluctuations (**right**) in the two considered intercalation modes (minor/major groove) of the DOX^…^dsDNA and 4-pep–DOX^…^dsDNA complexes. In addition, 100 overlaid representative positions of the protonated amino group (in the amino sugar of DOX or at the N-terminus of the tetrapeptide of 4-pep–DOX) are marked with spheres.

Figure 11 shows, separately, the most likely structures of DOX^…^dsDNA and 4-pep–DOX^…^dsDNA complexes identified by our cluster analysis for each of the four intercalation cases considered. As expected, the energetically most favorable DOX^…^dsDNA complex in the minor-groove mode is characterized by a well-defined structure with one preferred arrangement of the ligand with respect to the dsDNA duplex (85% of the bound ensemble). This is also manifested by the limited structural fluctuations of the ligand within the intercalation site, and by strong interactions between the positively charged amino group of DOX with dsDNA phosphate groups, with the mean distance to the closest phosphate being 0.62 nm. By comparison, intercalation of DOX from the major-groove side does not allow for a favorable electrostatic contact between the amino group and the dsDNA backbone (the mean distance to the closest phosphate is 1.06 nm), resulting in a substantially reduced stability of the complex.

In contrast to the DOX case, the most likely arrangement of 4-pep–DOX intercalated into dsDNA from the minor groove accounts for only 35% of the ensemble (Figure 11). In this position, the tetrapeptide fragment resides outside the minor groove, not interacting specifically with the dsDNA chain despite a relatively small average distance to the closest phosphate group (0.76 nm). Notably, DOX and 4-pep–DOX intercalated via the minor groove differ substantially in the orientation of their planar chromophore relative to the flanking base pairs. In particular, the 4-pep–DOX core is rotated by 33° resulting in a decreased stacking overlap with the flanking nucleobases, as compared to DOX (see Figure S3, Supplementary Materials).

In its more stable major-groove intercalation mode, 4-pep–DOX exhibits one well-defined structure (74% of the ensemble, Figure 11), in which the tetrapeptide interacts largely with one dsDNA strand, with the mean distance of the amino group to the closest phosphate equal to 0.45 nm. This suggests that effective stabilization of the intercalation complex requires accommodation of the flexible peptide substituent in the wider and more accessible major groove.

## 3. Materials and Methods

### 3.1. Materials

Fmoc–Leu–Ala–Gly–Gly and Fmoc–Gly–Pro–Leu–Gly–Leu–Ala–Gly–Gly peptides were purchased from Lipopharm.pl (Zblewo, Poland). Oligonucleotides 5′-CGT ACG CGT ACG CGT ACG CG-3′ and 5′-CGC GTA CGC GTA CGC GTA CG-3′ were purchased from Future Synthesis (Poznań, Poland). Collagenase IV and Hank’s Balanced Salt Solution (HBSS), with calcium and magnesium, were purchased from ThermoFisher Scientific (Walthman, MA, USA). Acetonitrile (ACN), 4-aminophenylmercuric acetate (APMA), dichloromethane (DCM), 2,5-dihydroxybenzoic acid (DHB), N,N-dimethylformamide (DMF), doxorubicin hydrochloride, formic acid, isobutyl chloroformate, methanol, sodium hydroxide, tetrahydrofuran (THF), triethylamine (TEA), and Tris-HCl were purchased from Sigma-Aldrich (St. Louis, MO, USA). Column chromatography was performed using silica gel NORMASIL 60 (40–63 mesh, VWR Chemicals, Radnor, PA, USA). Thin-layer chromatography was performed using silica plates, 60G, F_254_ (Sigma-Aldrich, St. Louis, MO, USA).

### 3.2. Methods

#### 3.2.1. Assembly of dsDNA

Two oligonucleotides of 20 base pairs, 5′-CGT ACG CGT ACG CGT ACG CG-3′ and 5′-CGC GTA CGC GTA CGC GTA CG-3′, were mixed at concentrations of 10 μM each, in 10 mM Tris-HCl buffer (pH 7.2). The mixtures were submitted to the annealing procedure at 95 °C for 5 min, 50 °C for 30 min, and cooled to 4 °C in 20 min using a Mastercycler gradient from Eppendorf (Hamburg, Germany). The formation of double-stranded DNA was confirmed by the HPLC Dionex Ultimate 3000 System (Waltham, MA, USA; data not shown). The analysis was performed on the XBridge OST C18 column (2.5 μm, 4.6 × 50 mm; Waters, Milford, MA, USA) at 20 °C. The flow was set at 1 mL/min with a mobile phase consisting of solvent A (1% ACN with 50 mM triethylamine acetate) and solvent B (80% ACN). The gradient was 0–20% of solvent B in 20 min.

#### 3.2.2. Enzymatic Cleavage of Peptide by MMPs

The MMP-2/-9-cleavable octapeptide Fmoc–Gly–Pro–Leu–Gly–Leu–Ala–Gly–Gly was evaluated by enzymatic digestion with the use of collagenase IV (containing MMP-2/-9). In the first step, 1 mL of HBSS was added to 1 g of collagenase IV (205 u/mg) and mixed to complete dissolution. Activation of collagenase IV was performed using 1 mM APMA in 0.15 M NaOH for 1.5 h at 37 °C [24,43]. The octapeptide stock solution was added to activated 1 mM collagenase IV and incubated at 37 °C. The aliquots were removed and analyzed by HPLC, between 2 and 12 h. The HPLC Dionex Ultimate 3000 (Waltham, MA, USA) was equipped with a bioZen Intact XB-C8 column (3.6 μm, 2.1 × 150 mm; Phenomenex, Aschaffenburg, Germany). The flow rate was set at 0.5 mL/min with a mobile phase consisting of solvent A (0.1% TFA) and solvent B (100% ACN). The gradient was as follows: 0–5 min, 0% of solvent B; 5–8 min, 0–30% of solvent B. The hydrolysis product was detected by MALDI TOF/TOF 5800 mass spectrometry (AB Sciex, Darmstadt, Germany).

#### 3.2.3. Synthesis of Fmoc–Leu–Ala–Gly–Gly–DOX

Isobutyl chloroformate (9.6 µL, 74.3 µmol) and TEA (10.4 µL, 74.3 µmol) were added to the solution of Fmoc–Leu–Ala–Gly–Gly (40 mg, 74.3 µmol) in DMF (1 mL) cooled to −10 °C. After 20 min of vigorous stirring, a solution of DOX (obtained by treating DOX hydrochloride (43 mg, 74.3 µmol) in DMF with TEA (10.4 µL, 74.3 µmol)) in DMF (0.5 mL) was added to the reaction mixture. After 1 h at RT, DMF was evaporated, azeotropically, with toluene on a rotary evaporator (Heidolph Hei-VAP Gold 3, Heidolph, Schwabach, Germany). The raw Fmoc–Leu–Ala–Gly–Gly–DOX was purified by preparative column chromatography, using CHCl3: MeOH 30: 1 as an eluent, to give the desired product (59 mg) in a 75% yield.

#### 3.2.4. Synthesis of Leu–Ala–Gly–Gly–DOX

Morpholine (0.5 mL) was added to a stirred solution of Fmoc–Leu–Ala–Gly–Gly–DOX (50 mg, 47 µmol) in DMF (0.5 mL), and the stirring was carried on for a further 2 h. Afterward, the mixture was neutralized with trifluoroacetic acid (0.6 mL). The raw product was isolated on a semi-preparative HPLC System (Shimadzu, LC 20AD, Shimadzu, Canby, OR, USA) using a Gemini NX Column (5.0 μm, 10 × 150 mm) to give 24 mg in a 61% yield. The flow rate was set at 4.0 mL/min with the mobile phase consisting of solvent A (0.1% formic acid) and solvent B (100% ACN). The gradient was as follows: 0–15 min, 10–90% of solvent B; 15–20 min, 90% of solvent B. The chemical structure of the product was confirmed using MALDI TOF/TOF 5800 mass spectrometry (AB Sciex, Darmstadt, Germany). As a matrix, 2,5-dihydroxybenzoic acid was used. The measurement was done in reflector positive ion mode. Samples were prepared using the dried droplet preparation method by mixing 0.8 mL of an analyte solution with 0.8 mL of the matrix solution (directly on a plate). MS spectra were acquired from 789 to 961 *m*/*z* for a total of 1000 laser shots by a 1 kHz OptiBeam laser (AB Sciex, Darmstadt, Germany).

#### 3.2.5. Spectrophotometric Titrations

The concentrations of the studied compounds (DOX and 4-pep–DOX) were confirmed spectrophotometrically based on the absorbance and the value of the molar extinction coefficient determined at 480 nm (ε480 = 11500 M^−1^ cm^−1^) [44]. UV-Vis spectrophotometric experiments were carried out in 10 mM Tris-HCl buffer (pH 7.2) at 25 °C using a PerkinElmer Lambda 650 UV/Vis spectrophotometer (Walthnam, MA, USA). UV-Vis absorption spectra of DOX and 4-pep–DOX (18.0 µM) were recorded in the absence and presence of increasing concentrations of dsDNA, up to 1.90 µM. In the performed spectrophotometric experiments, 2 mL of DOX and 4-pep–DOX at 18.0 µM were titrated with ten 10 μL aliquots of dsDNA solution at 40 µM.

#### 3.2.6. Steady-State and Time-Resolved Fluorescence Spectroscopy

Steady-state fluorescence experiments were carried out at 25 °C using a Cary Eclipse Varian spectrofluorometer (Agilent, St. Clara, CA, USA) equipped with a temperature controller and a 1.0 cm quartz multicell holder. The fluorescence emission spectra (λ_ex_ = 490 nm) of DOX and 4-pep–DOX (1.94 µM) were recorded from 510 to 690 nm in the absence and presence of increasing concentrations of dsDNA, up to 75 nM. In the performed fluorescence titration experiments, 2 mL of DOX and 4-pep–DOX at 1.94 µM were titrated with fifteen 1 μL aliquots of dsDNA solution at 10 µM. The intensity of the band at 594 nm (corresponding to the maximum emission of both, DOX and its analogue 4-pep–DOX) was used to calculate the association constants (K_A_) and other parameters. Time-resolved fluorescence measurements were performed with a FluoTime 300 high performance fluorescence lifetime spectrometer (PicoQuant, Berlin, Germany) at 20 °C. The excitation source was a pulsed LED of the PLS series (λex = 420 nm). The fluorescence lifetimes of both DOX and 4-pep–DOX (5 µM) were measured in the absence and presence of variable concentrations of dsDNA (0.05 µM, 0.10 µM, 0.15 µM, 0.20 µM, and 0.25 µM). After each added portion of dsDNA, the studied solution was gently stirred, and the fluorescence lifetime was measured at the wavelength corresponding to the maximum of the fluorophore emission (λ_em_ = 594 nm).

#### 3.2.7. Isothermal Titration Calorimetry

All ITC experiments were performed at 25 °C using an AutoITC isothermal titration calorimeter (MicroCal Inc. GE Healthcare, Northampton, MA, USA). The details of the measuring devices and experimental setup have been described previously [45]. The reagents (DOX, 4-pep–DOX, and dsDNA) were dissolved directly in 10 mM Tris-HCl buffer solution (pH 7.2). The experiment comprised injections of 10.02 μL (29 injections, 2 μL for the first injection only) of the buffered solution of DOX (0.174 mM) or 4-pep–DOX (0.470 mM) into the reaction cell, which initially contained the buffered solution of dsDNA (0.002 mM or 0.005 mM, respectively). For each experiment, a blank was performed by injecting the titrant solution into the cell filled with the buffer only. This blank was subtracted from the corresponding titration to account for the heat of dilution. All solutions were degassed prior to the titration. The titrant was injected at approximately 4 min intervals to ensure that the titration peak returned to the baseline before the next injection. Each injection lasted 20 s. To ensure a homogeneous mixing in the cell, the stirrer speed was kept constant at 300 rpm. Calibration of the AutoITC calorimeter was carried out using electrically generated heat pulses. The CaCl_2_-EDTA titration was performed to check the apparatus and the results (*n*–stoichiometry, K, Δ*H*) were compared with those obtained for the same samples (a test kit) at MicroCal Inc./Malvern Instruments (Malvern, England).

#### 3.2.8. Molecular Dynamics Simulations

All simulated systems were built on the basis of the crystal structure of the doxorubicin–dsDNA complex (PDB id 1D12) [46]. Initial coordinates of a 20-bp long dsDNA helix with 5′-CGT ACG CGT ACG CGT ACG CG-3′ sequence were generated by removing the two base pairs at the 3′-end of dsDNA in the crystal structure, and then the appropriate extension of the structure at the 5′ and 3′ ends by using the X3DNA program [47]. The obtained dsDNA–drug complexes were solvated with 13,947 TIP3P water molecules in a dodecahedral box with a cell vector length of 8.5 nm, at physiological ionic strength (150 mM NaCl) [48]. The Amber parmbsc1 force field was used for dsDNA and ions [49]. The force field parameters for DOX and the tetrapeptide fragment of 4-pep–DOX were taken from the General Amber Force Field (GAFF) and directly from Amber, respectively [50]. Partial charges for both ligands were obtained from HF calculations in Gaussian via Merz–Kollman ESP fitting, using the 6-31G* basis set [51]. The MD simulations were performed using Gromacs 5.0.4 in the NPT ensemble, with the temperature kept at 25 °C and using the v-rescale thermostat with the pressure kept at 1 bar using a Parrinello–Rahman barostat [52,53,54]. Periodic boundary conditions were applied in 3D, and the electrostatic interactions were calculated using the particle mesh Ewald (PME) method with a real-space cut-off of 1.2 nm and a Fourier grid spacing of 0.12 nm [55]. A cut-off of 1.2 nm was used for Lennard-Jones interactions. All bond lengths were constrained using P-LINCS for dsDNA and SETTLE for water [56,57]. The equations of motion were integrated using the leap-frog algorithm with a 2 fs time step.

To study the relative stability of the complexes formed by DOX and 4-pep–DOX with dsDNA duplex in two different intercalation modes (with the amino sugar extending into either the minor or major groove), we calculated the corresponding free energy profiles using replica-exchange umbrella sampling (REUS). The calculations were carried out using the PLUMED 2.0 plugin coupled to Gromacs [58]. To generate the initial configurations for the REUS simulations, we first performed short equilibrium simulations (150 ns) of the DOX and 4-pep–DOX, initially intercalated at a CpG step of dsDNA, in either a minor- or major-groove mode. In the former case, the initial configuration of the DOX^…^dsDNA intercalation complex was taken directly from the crystal structure and the 4-pep–DOX^…^dsDNA complex was then obtained by attaching the tetrapeptide moiety to the amino group of DOX. In the latter case, the orientation of both intercalators was manually inverted within the same intercalation site. To obtain the dissociated states for the so-prepared four systems, we ran steered MD (SMD) simulations, in which the ligands were pulled away from dsDNA during 100 ns using a moving harmonic potential with a force constant of 286.1 kcal/(mol·nm^2^) applied to the coordinate, defined as the separation distance between the centers of mass of the two base pairs forming the intercalation site and the planar chromophore of DOX and 4-pep–DOX (r vector in Figure 10A).

Next, from SMD trajectories, we generated 21 uniformly distributed 0.2 nm separated REUS windows. In each of these windows, the systems were simulated for 0.5 µs using the harmonic potential with a force constant of 143.05 kcal/(mol·nm^2^) to restrain the system along the reaction coordinate r. In addition, we used one-sided harmonic potentials with a force constant of 286.1 kcal/(mol·nm^2^), turning on below 1.2 nm to prevent ligands binding at the dsDNA ends (grey dashed lines in Figure 10A). The exchanges between neighboring windows were attempted every 2 ps, and the acceptance rate turned out to be 19 %. The free energy profiles were determined from the last 450 ns of the thus obtained trajectories using the standard weighted histogram analysis method [59]. All molecular images were created using VMD 1.9.2 (Univerisity of Illinois, Urbana, IL, USA) [60].

## 4. Conclusions

In this work, we successfully synthesized the tetrapeptide-DOX (4-pep–DOX) adduct as a product of octapeptide cleavage by MMPs, for extensive investigation of the parameters that are critical in forming a 4-pep–DOX^…^dsDNA complex. The experimental and theoretical studies demonstrated that the presence of tetrapeptide does not affect the overall tendency of DOX to intercalate into dsDNA, but rather changes its binding mode. While the main mode of action of DOX has been attributed to its intercalation from the minor groove of dsDNA, the binding of 4-pep–DOX primarily occurs from the major groove. Multifaceted binding modes for DOX and 4-pep–DOX highlight the important role of electrostatic and hydrophobic interactions between the negatively charged phosphate group on the groove surface and the peptide chain of 4-pep–DOX. Hence, these results demonstrate that the release of a free DOX from the MMP-cleaved intermediate product by intracellular non-specific proteases is probably not vital to the cytostatic action of the drug. We believe that the presented detailed explanation of the intercalation mechanism of 4-pep–DOX adduct can be useful for paving the way for the future design of peptide–DOX prodrugs.

## Figures and Tables

**Table 1:** Thermodynamic parameters of DOX and 4-pep–DOX bound to dsDNA in 10 mM Tris-HCl buffer (pH 7.2) at 25 °C (standard deviation values in parentheses). ITC, isothermal titration calorimetry.

Parameter	DOX^…^dsDNA	4-pep–DOX^…^dsDNA
log*K_ITC_*	6.03 (±0.04)	4.24 (±0.09)
Δ*H_ITC_* [kcal/mol]	−9.52 (±0.17)	−5.84 (±1.27)
TΔ*S_ITC_* [kcal/mol]	−1.3	−0.05
Δ*G_ITC_* [kcal/mol]	−8.26 (±0.05)	−5.78 (±0.12)

## Supplementary Materials

Supplementary materials can be found at https://www.mdpi.com/1422-0067/21/18/6923/s1.

## Abbreviations

DOX	Doxorubicin
MMPs	Matrix metalloproteinases
MD	Molecular dynamics
ITC	Isothermal titration calorimetry
