# Supplementary material for: The Product of Matrix Metalloproteinase Cleavage of Doxorubicin Conjugate for Anticancer Drug Delivery: Calorimetric, Spectroscopic, and Molecular Dynamics Studies on Peptide–Doxorubicin Binding to DNA
Source: Int J Mol Sci. 2020 Sep 21;21(18):6923. doi: 10.3390/ijms21186923 (PMC7554696; doi:10.3390/ijms21186923)
Supplement: Supplementary file 1 [file ijms-21-06923-s001.pdf]

Supplementary data

The product of matrix metalloproteinase cleavage of doxorubicin conjugate for anticancer drug delivery. Calorimetric, spectroscopic and molecular dynamics studies on peptide–doxorubicin binding to DNA

Kamila Butowska [1,2,*], Krzysztof Żamojć [3], Mateusz Kogut [4], Witold Kozak [2], Dariusz Wyrzykowski [3], Wiesław Wiczk [5], Jacek Czub [4], Jacek Piosik [1] and Janusz Rak [2]

[1] Laboratory of Biophysics, Intercollegiate Faculty of Biotechnology University of Gdańsk and Medical University of Gdańsk, Abrahama 58, 80-307 Gdańsk; kamila.butowska@phdstud.ug.edu.pl (K.B.); jacek.piosik@biotech.ug.edu.pl (J.P.)

[2] Department of Physical Chemistry, Faculty of Chemistry, University of Gdańsk, Wita Stwosza 63, 80-308 Gdańsk; davelombardo@wp.pl (W.K.); janusz.rak@ug.edu.pl (J.R.)

[3] Department on General and Inorganic Chemistry, Faculty of Chemistry, University of Gdańsk, Wita Stwosza 63, 80-308 Gdańsk; krzysztof.zamojc@ug.edu.pl (K.Z.); dariusz.wyrzykowski@ug.edu.pl (D.W.)

[4] Department of Physical Chemistry, Faculty of Chemistry, Gdańsk University of Technology, Narutowicza 11/12, 80-233 Gdańsk; giggsmk@op.pl (M.K.); jacek.czub@pg.edu.pl (J.C.)

[5] Department of Biomedical Chemistry, Faculty of Chemistry, University of Gdańsk, Wita Stwosza 63, 80-308 Gdańsk; wieslaw.wiczk@ug.edu.pl (W.W.)

* Correspondence: kamila.butowska@phdstud.ug.edu.pl; Tel.: +48 58 523 6310

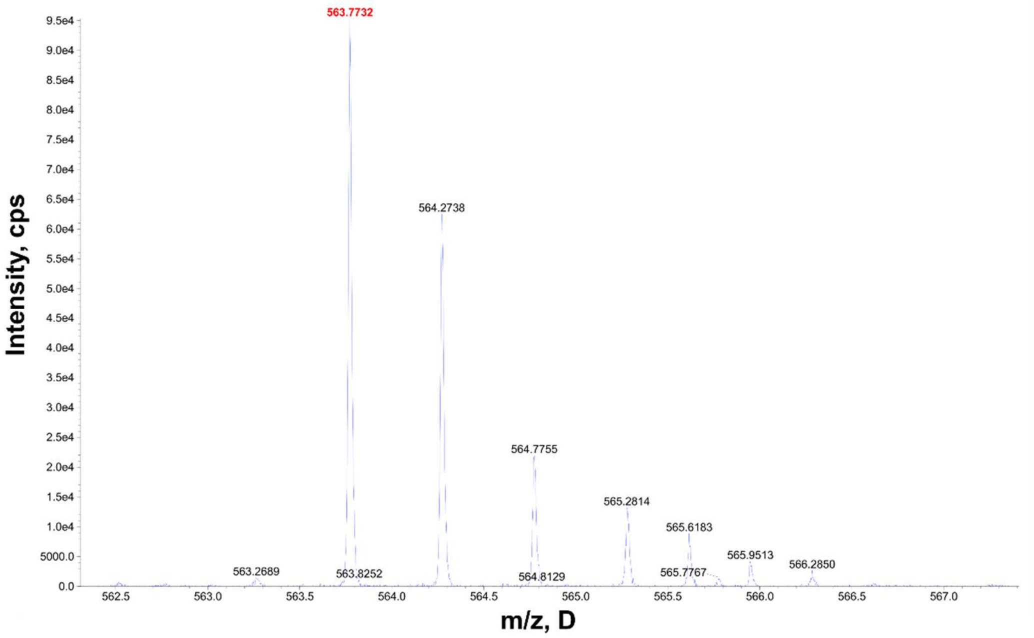

Figure S1. MALDI-TOF mass spectrum of Fmoc–Gly–Pro–Leu–Gly in a positive mode (m/z 563.7732).

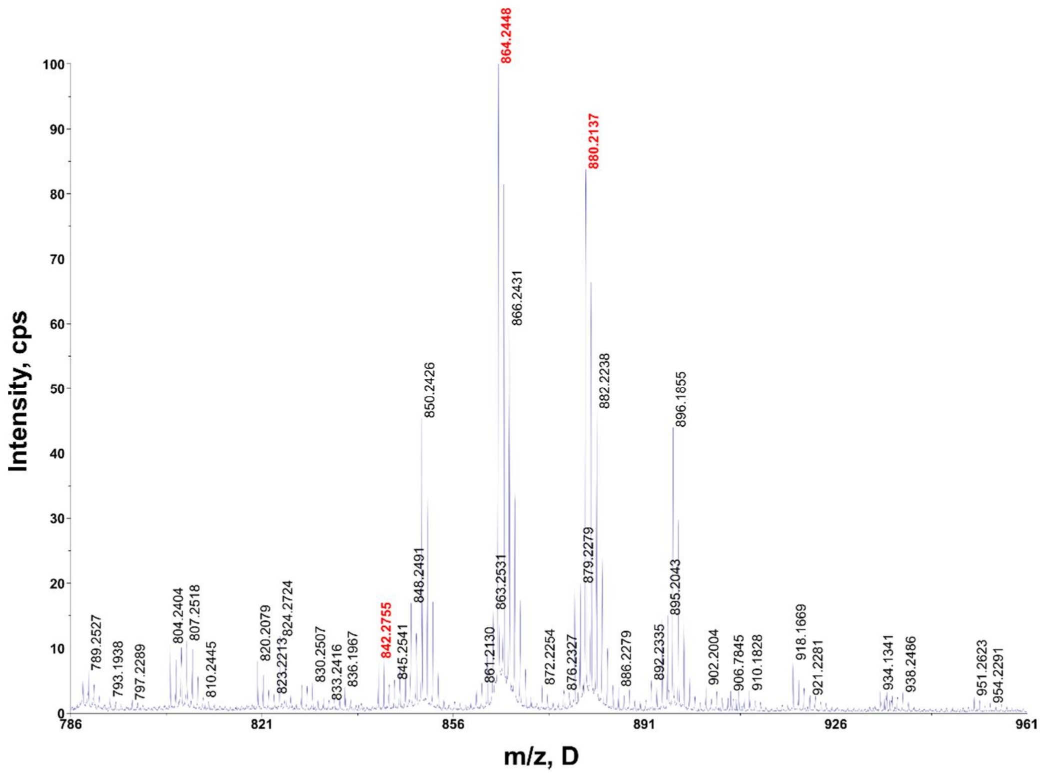

Figure S2. MALDI-TOF spectrum of Leu–Ala–Gly–Gly–DOX in a positive mode [(Leu–Ala–Gly–Gly–DOX + H^+) m/z 842.2755; (Leu–Ala–Gly–Gly–DOX + Na^+) m/z 864.2448; (Leu–Ala–Gly–Gly–DOX + K^+) m/z 880.2137].

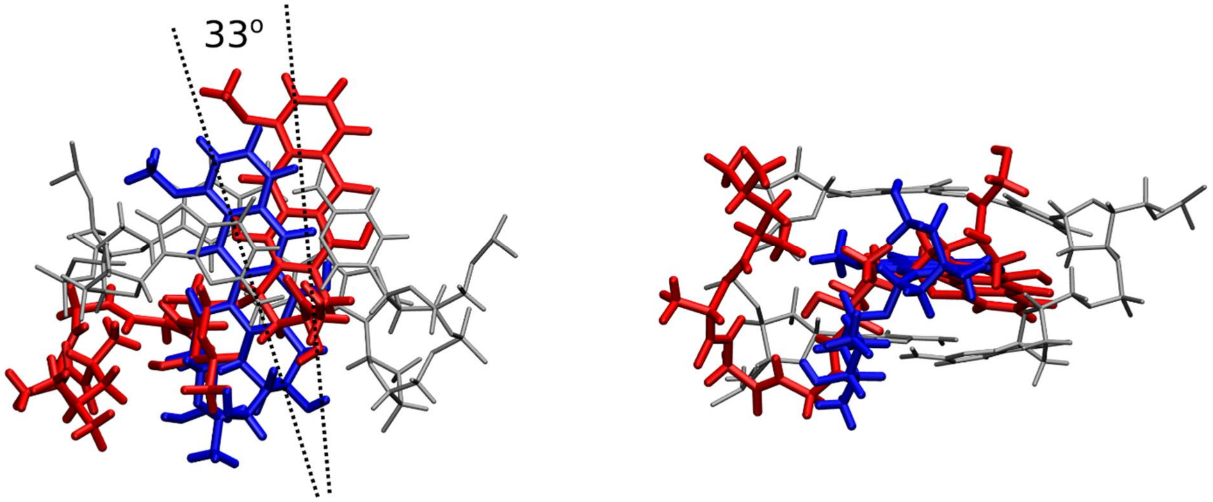

Figure S3. Comparison of the minor groove intercalation modes of DOX (blue) and 4-pep-DOX (red) -- top view (left), side view (right). When intercalated from the minor groove, 4-pep-DOX is rotated by ca. 33deg with respect to the flanking base pairs, compared to DOX.
